# Prevalence of hepatitis E virus and reassessment of HIV and other hepatitis virus seroprevalences among French prison inmates

**DOI:** 10.1371/journal.pone.0218482

**Published:** 2019-06-26

**Authors:** Laure Izquierdo, Guillaume Mellon, Céline Buchaillet, Catherine Fac, Marie-Pierre Soutière, Coralie Pallier, Anne Dulioust, Anne-Marie Roque-Afonso

**Affiliations:** 1 Virologie, Hôpital Paul Brousse, AP-HP, Villejuif, France; 2 INSERM U1193, Villejuif, France; 3 Université Paris-Sud, Bicêtre, France; 4 Médecine, Etablissement Public National de Santé de Fresnes (EPSNF), Fresnes, France; 5 Maladies Infectieuses et Tropicales, Hôpital Saint Louis, AP-HP, Paris, France; 6 Unité de Consultations et de Soins en Ambulatoire (UCSA), Centre Pénitentiaire de Fresnes, Centre Hospitalier Universitaire de Bicêtre, Le Kremlin-Bicêtre, France; Centre de Recherche en Cancerologie de Lyon, FRANCE

## Abstract

**Background:**

Prison inmates are considered a high-risk population for blood-borne and enterically transmitted infections before and during their imprisonment. Hepatitis E virus (HEV) prevalence is unknown among French inmates, whereas a reassessment of human immunodeficiency virus (HIV), hepatitis A virus (HAV), hepatitis B virus (HBV) and hepatitis C virus (HCV) prevalences is required to describe the epidemiologic evolution in this high-risk population.

**Methods:**

A prospective survey was conducted from June to December 2017 in Fresnes prison, a penitentiary center with 2,581 inmates. In addition to HIV, HAV, HBV and HCV testing, which is offered to all patients at admission, we systematically offered HEV screening. Retrospective serological data for HIV, HBV and HCV, collected annually from 2014 to 2017, were also used to assess evolution.

**Results:**

In 2017, 1,093 inmates were screened for HEV, HIV, HAV, HBV and HCV. Prevalences in this population were 8.2%, 1.3%, 62.7%, 1.9% and 2.9%, respectively. HEV seroprevalence increased with age (p<0.0001) and was higher among Eastern Europe born inmates (p<0.0001). Between 2014 and 2017, HIV seroprevalence remained steady, while a decrease in HBV and HCV seroprevalence was observed.

**Conclusions:**

Compared to the reported prevalence in French blood donors, HEV seroprevalence was remarkably low in French inmates. HIV, HAV, HBV and HCV prevalences among prisoners were higher than reported in the general population.

## Introduction

Infectious diseases have been shown to be more prevalent among precarious individuals than in the general population. Before incarceration, low socioeconomic status and poor hygienic standards are common characteristics of future inmate patients, and this could contribute to higher exposure to enterically transmitted viruses such as hepatitis E virus (HEV). HEV is known to be a food and water‐borne disease, responsible for mostly asymptomatic infection and classically cytolytic hepatitis.

Otherwise, inmates are considered a high-risk population for blood-borne and sexually transmitted infections such as human immunodeficiency virus (HIV), hepatitis B virus (HBV) and hepatitis C virus (HCV) due to high transmission risk behaviors before incarceration, including injecting drug use or sex work for example. Prison represents a high-risk environment of acquiring viral infections due to the promiscuity and the continuation of behaviors with high transmission risk [1–3]. Thus, the inmate population potentially constitutes either a reservoir or an observatory for epidemiological changes. Because of these risks, the French National Authorities for Health recommend offering HIV, HBV and HCV screening at prison’s admission, as well as repeating this offer periodically during detention [4]. Compared to the general population, little data exist on the prevalence of HEV infection among inmates population [5]. Furthermore, while prevalences of HIV, HAV, HBV and HCV are available, the reports are scattered and offer an incomplete assessment [1,3,6].

In this study, we aimed to determine the HEV seroprevalence among inmate patients and to perform a reappraisal of the HIV, HAV, HBV and HCV data for this high-risk population.

## Materials and methods

### Study population

This prospective survey was conducted between June and December 2017 in Fresnes prison. Fresnes is the second largest French penitentiary center housing 2,581 (3.7%) inmates of the 69,714 prisoners held in prison in France on December 2017.

### Ethics approval and consent to participate

No formal ethics approval was required for the present study, in accordance with the Jardé law concerning research involving human persons (order 2016–800 of June 2016, 16th), since this study focuses on data collected as part of patient’s care. However, it falls within opinion of the French National Commission for Information Technology and Liberties (Commission Nationale de l’Informatique et des Libertés—CNIL). Data collected during the current study were analyzed in a folder declared to the CNIL under registration number 2170170. All patients gave their oral informed consent for sample and data collection. Oral consent was documented in each individual medical file by physicians in charge of the inmates at the admission visit. Written consent was not necessary because the samples drawn were part of the patient’s care. No samples nor data were taken or collected for patients who did not accept to be tested.

### Data collection

All inmates, at admission to this center, are systematically offered HIV, HAV, HBV, HCV, syphilis, chlamydia and gonorrhea testing. For the present study, HEV screening was also systematically offered from June to December 2017. Blood samples were taken after informed consent. A retrospective review of the prisoner’s medical files offered serological data for HIV, HBV and HCV status from 2014 to 2017.

### Serological assays

Samples were analyzed for anti-HEV immunoglobulin G (IgG) and M (IgM) using the Wantai HEV-IgG ELISA and HEV-IgM ELISA kits (Wantai Biologic Pharmacy Enterprise, Beijing, PRC). Samples were tested for HIV, HAVand HCV antibodies, hepatitis B surface antigen (HBsAg), hepatitis B surface antibodies (anti-HBs) and hepatitis B core antibodies (anti-HBc) using the HIV combi PT, Anti-HAV, Anti-HCV II, HBsAg II, Elecsys Anti-HBs II and Elecsys Anti-HBc II electrochemiluminescence immunoassays kits (Cobas Roche Diagnostics, Rotkreuz ZG, Switzerland). In case of positive HBsAg, clinical samples were also tested for HDV antibodies with the ETI-AB-DELTAK-2 enzyme-linked immunosorbent assay (Diasorin, Saluggia VC, Italy).

### HIV-1, HBV and HCV quantification

All samples reactive for HIV, HBsAg and anti-HCV were analyzed for HIV-1 RNA, HBV DNA and HCV RNA using the Abbott *m*2000 RealTi*m*e system (Abbott Molecular, Chicago, IL).

### HCV genotyping

HCV genotyping was performed by sequence analysis of a 380-nucleotide fragment of the NS5B region, as previously described [7].

### Statistical analysis

Prevalence differences were analyzed using univariate analyses. Student’s *t*, Fisher’s exact and Chi-squared tests were used as appropriate. Statistical significance was defined as p-values <0.05. Statistical analysis was performed using Analyse-it version 5.11 / 2.30 (Win) / 2018 for Windows.

## Results

During the study period, 2,167 individuals were admitted to the center. We performed 1,168 anti-HEV tests, corresponding to a 53.9% HEV screening rate. Among them, 1,093 inmates with concomitant HIV, HAV, HBV and HCV screening were selected for the study. The mean age was 31 years (range 18–80) and included 982 (89.8%) men and 111 (10.2%) women. Among the entire cohort, the proportion of foreign birth prisoners was 56%. Western Europe born prisoners (including France) accounted for 46.6%, followed by prisoners born in Middle East, North Africa and Greater Arabia 19.3%, those born in Central and South America 12.2%, in Sub-Saharan Africa 11.9%, in Eastern Europe 7.8%, and finally in Asia 2.1%. There was no significant variation of inmates’ mean age according to region of birth.

### HEV prevalence

**Fig 1:**
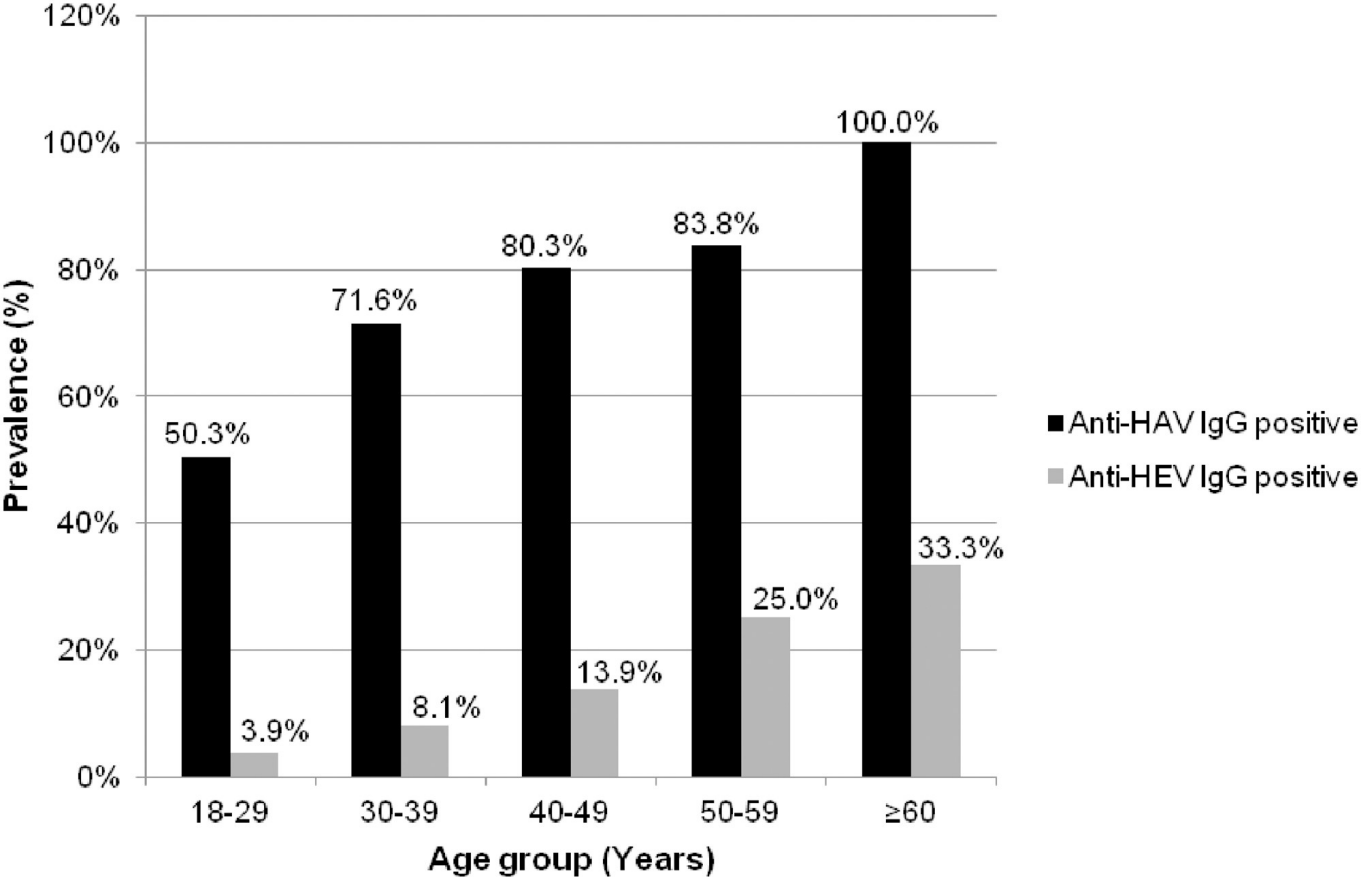
Prevalences of anti-HAV IgG and anti-HEV IgG by age group.

No HEV IgM was detected in our trial. HEV seroprevalence was 8.2% and did not differ between women and men (10.8% and 7.9%, respectively), but increased with age from 3.9% in the age group 18–29 years to 33.3% in the age group ≥60 years (p<0.0001) (Fig 1). Of the 90 HEV-seropositive prisoners, 18 (20%) and 36 (40%) used to eat pork meat and berries, respectively. Unfortunately, dietary habits were not recorded for the whole population and these factors could not be tested as associated with HEV positivity. There was no association between HEV positivity and HIV, HBsAg or HCV positivity. Compared to HEV-negative subjects, HIV seroprevalence among HEV-positive subjects was 1.1% *vs*. 1.3% (p = ns), HBsAg seroprevalence was 2.2% *vs*. 1.9% (p = ns), and HCV seroprevalence was 3.3% *vs*. 2.9% (p = ns). However, as shown in Table 1, HEV prevalence was significantly higher among inmates born in Eastern Europe than among those born in other regions (p<0.0001).

**Table 1:** HEV prevalence per birth’s region.

Factor	N° Samples tested	% of HEV IgG positive	P*
**Gender**			0.2776
Male	982	7.9%	
Female	111	10.8%	
**Age Group (years)**			<0.0001
18–29	590	3.9%	
30–29	271	8.1%	
40–49	137	13.9%	
50–59	68	25%	
60+	27	33.3%	
**Birth Region**			<0.0001
Asia	23	8.7%	
Central and South America	133	4.5%	
Eastern Europe	85	22.4%	
Mainland France	479	4.8%	
Middle East, North Africa, and Greater Arabia	210	11.9%	
Sub-Saharan Africa	130	10%	
Western Europe	29	6.7%	
		

*Pearson Chi square test

### HIV, HAV, HBV and HCV prevalences

**Fig 2:**
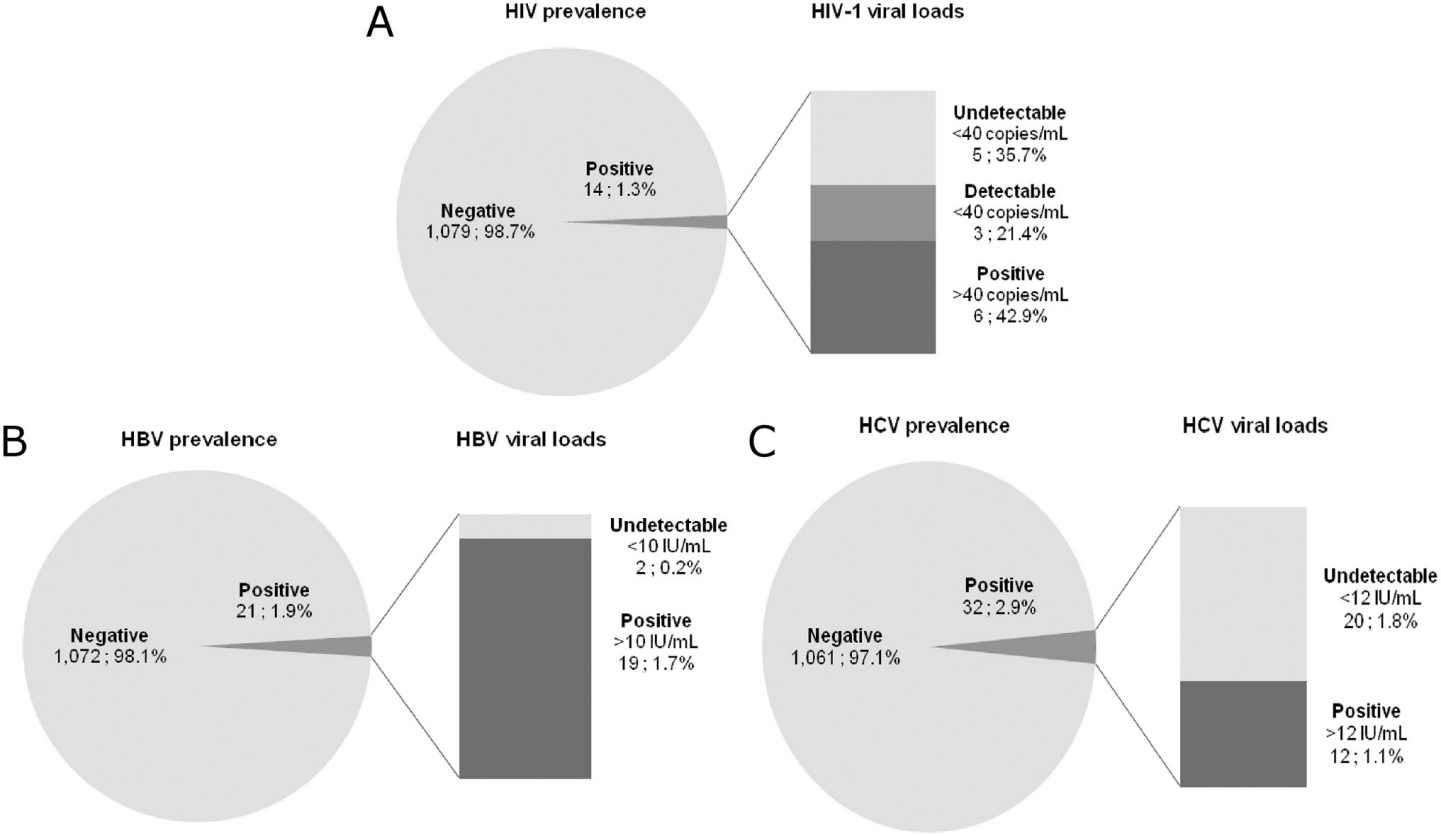
Viruses prevalences and quantification. (A) HIV prevalence and viral quantification. (B) HBV prevalence and viral quantification. (C) HCV prevalence and viral quantification.

Fig 2A shows HIV prevalence, which is unrelated to sex, but the HIV-seropositive individuals were older than the HIV-seronegative ones (45.0 [36.2–53.8]_95% CI_ *vs*. 31.3 [20.3–42.3]_95% CI_, p<0.0001). Of the participants with a HIV-1 RNA quantification (Fig 2A), 50% had been diagnosed during imprisonment and 50% were already known as HIV-seropositive. All the 14 prisoners living with HIV received anti-retroviral therapy.

HAV seroprevalence was 62.7%, not related to sex, but increased with age from 50.3% in inmates younger than 29 years to 100% among those older than 60 years (p<0.0001) (Fig 1).

Fig 2B shows HBV prevalence, we did not notice differences in respect to sex or age. All participants were HDV antibody negative. Fig 2B represents HBV load with a mean viral load of 10,513,784 IU/mL (range 22–199,695,710 IU/mL). Eight inmates had HBV DNA quantification of more than 2,000 IU/mL, but only one was currently receiving treatment. In addition, 449 (41.1%) had HBV serology compatible with HBV vaccination, 114 (10.4%) with past HBV infection and 478 (43.7%) had no evidence of contact with HBV and were eligible to HBV vaccination.

Fig 2C displays HCV seroprevalence. The HCV-seropositive inmates were older than the HCV-seronegative ones (44.8 [51.7–37.9]_95% CI_ *vs*. 31.1 [20.1–42.1]_95% CI_, p = 0.002). HCV genotypes were G1a (n = 6), G3a (n = 5) and G4a (n = 1). Among the 12 HCV RNA-positive inmates (Fig 2C), seven (58.3%) had received or were receiving direct-acting antiviral treatment and five (41.7%) were released from prison without any treatment. The prevalence of HIV-HCV co-infection was 0.4% (4/1093).

### HIV, HBV and HCV prevalences over four years

**Fig 3:**
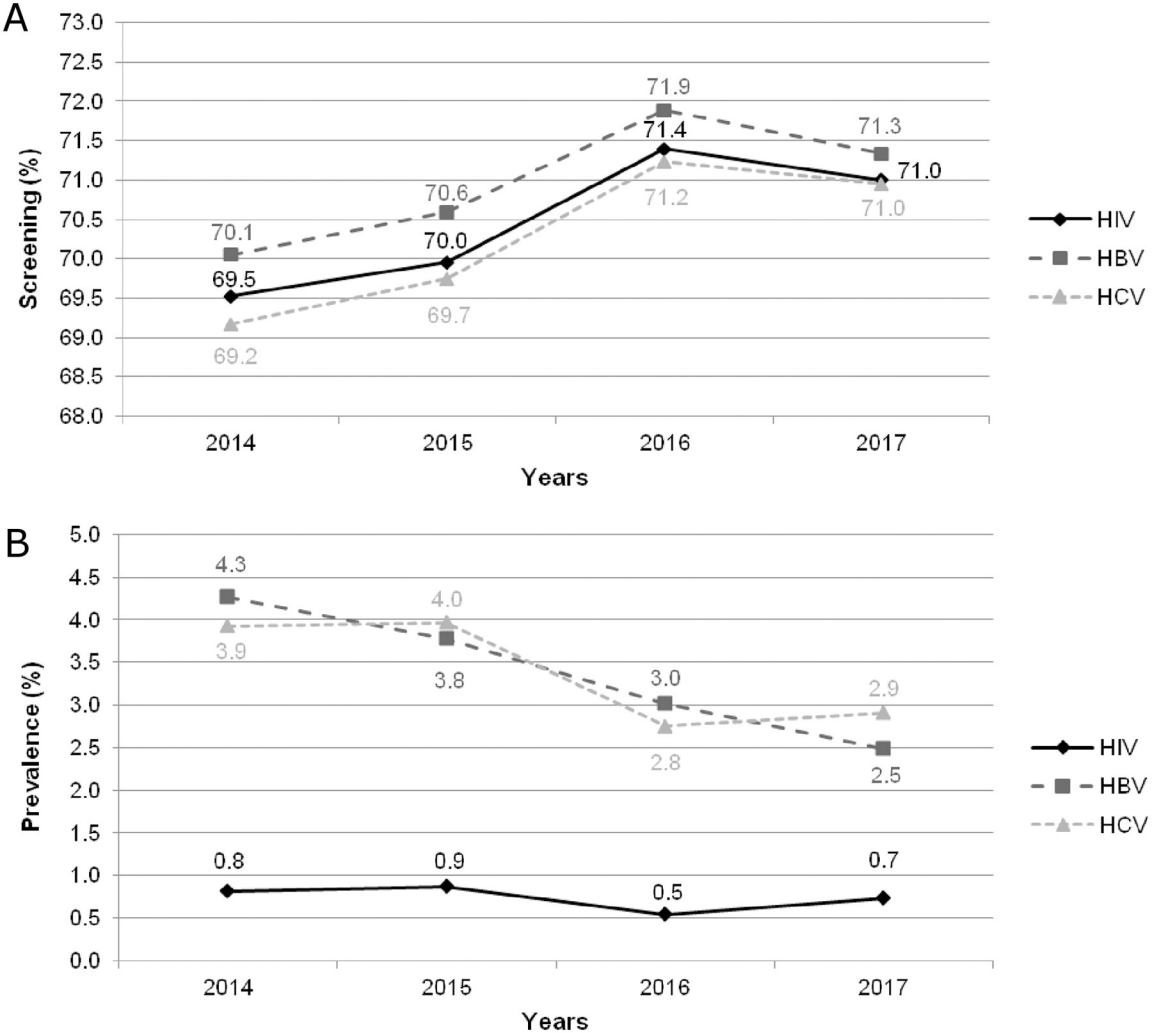
Evolution of viruses screening rate and prevalences from 2014 to 2017. (A) Screening rate. (B) HIV, HBV and HCV prevalences.

The yearly screening acceptance rate at prison’s admission remained steady at around 70% between 2014 and 2017 (Fig 3A). HIV prevalence remained stable over time, at less than 1%, as shown in Fig 3B. However, HBV and HCV prevalences significantly decreased during this same period (4.3% *vs*. 2.5%, p = 0.0019 and 3.9% *vs*. 2.9%, p = 0.016).

## Discussion

In our study, we estimated an anti-HEV IgG prevalence of 8.2%. We did not report any presentation of acute hepatitis E. The prevalence found in this study is the first report from a French prison and complements a previous report from inmates in Italy [5]. Notably, the HEV seroprevalence was lower than the 22.4% prevalence reported among French adult blood donors in 2011–2012 [8]. Though the median age in this latter prevalence study was higher than in the present study (45 *vs*. 31 years), the prevalence by age group among French blood donors appeared higher than among inmates with 10.5% for age group 18–27 and 14% for age group 28–37 *vs*. 3.9% and 8.1% reported in the present study among French inmates. Over 70% of the HEV-seropositive participants were of foreign birth, even though foreign-born inmates represented only 56% of our entire cohort. Inmates born in Eastern Europe had a higher HEV seroprevalence (22.4%) than those born in other regions, including mainland France. This finding is in line with an Italian study which reported higher HEV prevalence in non-Italian compared to Italian inmates [5]. This also suggests a remarkably low HEV seroprevalence in French inmates compared to the general population, possibly attributable to differences in lifestyle and diet, and possibly religious beliefs. Unfortunately, for ethical reasons, this information was not available. Consistent with previous reports conducted in different populations, HEV seroprevalence increased with age, possibly reflecting a lifelong cumulative risk of exposure to HEV [5,8,9]. Finally, we found no statistical association between HEV positivity and HIV, HBsAg or HCV positivity.

Moreover, this serological survey provides trends on HIV, HBV and HCV prevalences evolution among Fresnes inmates. A significant decrease in HBV and HCV prevalences was observed between 2014 and 2017. The decline of HCV prevalence in the prison setting has already been observed in a previous survey conducted in southeastern France between 2004 and 2010 (from 7.9% to 3.5%, respectively) [3]. In the present study, the decreasing HBV and HCV prevalences were not associated with a decrease in screening acceptance rate during the same period, with the annual screening rate remaining stable around 70% from 2014 to 2017. This response rate is consistent with published rates [6]. Our findings confirm previous reports, HIV, HBV and HCV prevalences were higher in the Fresnes prison population than reported in the general population [3,6,10,11]. HIV prevalence in the French general population was estimated at 0.40% in 2013 [12]. HBV and HCV prevalences were estimated in 2004 at 0.65% and 0.84%, respectively [13]. We also noticed that HIV and HCV prevalences observed in Fresnes in 2017 were lower than previously reported among prisoners in France and in most countries worldwide, where epidemiological situations are recognized as heterogeneous. Global HIV prevalence estimates in inmates, published between 2005 and 2015, were 3.8% [11] and the Prevacar survey, conducted in 2010 in 27 French prisons, indicated a prevalence of 2.0% [6]. Among people incarcerated worldwide in 2014, HCV prevalence was estimated at 15.1% [11] but varied widely in the European Union with estimates ranging from 4.3% to 86.3% between 2005 and 2015 [14]. In France, recent studies reported HCV prevalence rates around 5% [3,10], confirmed by the Prevacar study at 4.8% [6]. For HBV, while HBsAg prevalence estimates were reported as 4.8% worldwide [11], a relatively low prevalence of 0.6% was reported in a French study conducted from 2012 to 2013 [10]. This is significantly lower than the prevalence observed in 2017 in our study. However, like HIV and HCV, there is considerable heterogeneity in HBsAg prevalence in the European prison population as is shown by Falla *et al*., ranging from 0.3% to 25.2% [14]. Of note, the demographic characteristics of our study population are like those published by the French penitentiary administration in 2015, with a majority (97%) of prisoners being male with a mean age of 34.6 years [6,15]. In these national statistics, around 20% were of foreign origin.

Finally, the 62.7% prevalence of anti-HAV antibodies in inmates was much higher than the 2010 estimate of 25.6% for all 6–49 years in the French general population. This high seroprevalence was consistent with published data from Italian and Portuguese inmate populations (86.4% and 69.5%, respectively) [5,16]. Among Fresnes inmates, as in the general population in France or in Italy, HAV seroprevalence increased with age [17].

Some limitations should be acknowledged for the present study. First, this survey was conducted in a single penitentiary center. Therefore, though the Fresnes prison population may be considered a representative inmate population, a national level study with a larger population would better account for regional variations in viral prevalence rates. Second, information associated with HIV and hepatitis transmission and high-risk behaviors (e.g. injecting drug use, blood exposure, tattooing, dietary factors) are lacking. Nevertheless, we believe our study contributes to the understanding of viral infections in a high-risk French population. The main strength of this study is that virological tests were systematically administered to more than a thousand inmates and we were able to prospectively test for seropositivity to identify point prevalence and changes in prevalence over time. Until now, the majority of descriptive studies have estimated prevalence from retrospective data reviewed from medical records.

## Conclusions

With the present study, a remarkably low HEV seroprevalence and a high HAV seroprevalence were observed. We provided updated information on HIV and viral hepatitis prevalences in the inmate population in France, with an observed decrease of HBV and HCV prevalences among Fresnes prison inmates between 2014 and 2017. This survey underlines the importance of screening for viral infections among inmates to promote access to care and treatment and to improve prevention and vaccination strategies. Since prevalence rates evolve over time, it is necessary to carry out these serological studies regularly to re-evaluate funding allocated to harm reduction programs in high-risk populations.

## Supporting information

S1 Table Participants included in the study. (XLSX)
